# Major immediate insertion-related complications after central venous catheterisation and associations with mortality, length of hospital stay, and costs: A prospective observational study

**DOI:** 10.1177/11297298231222929

**Published:** 2024-01-24

**Authors:** Sofia Ingefors, Maria Adrian, Gawain Heckley, Ola Borgquist, Thomas Kander

**Affiliations:** 1 Medical Faculty, Lund University, Lund, Sweden; 2 Anaesthesiology and Intensive Care, Department of Clinical Sciences Lund, Lund University, Lund, Sweden; 3 Department of Cardiothoracic Surgery, Anaesthesia and Intensive Care, Skåne University Hospital Lund, Sweden; 4 Health Economics Unit, Department of Clinical Sciences, Lund University, Lund, Sweden; 5 Department of Intensive and Perioperative Care, Skåne University Hospital Lund, Sweden

**Keywords:** Central venous catheter, immediate insertion-related complication, length of hospital stay, medical costs, mortality

## Abstract

**Background:**

It is well-known that infectious complications after central venous catheterisation are associated with increased mortality, length of hospital stay and costs. However, there are limited data regarding such associations for immediate insertion-related complications. Therefore, the aim of this study was to investigate whether major immediate insertion-related complications are associated with mortality, length of hospital stay and costs.

**Methods:**

This was a preplanned substudy to the CVC-MECH trial on immediate insertion-related complications after central venous catheterisation in the ultrasound-guided era. Patients receiving central venous catheters at Skåne University Hospital from 2 March 2019 to 31 December 2020 were prospectively included. Patient characteristics, clinical data and costs were automatically collected from medical journals and the patient administration system. Associations between major immediate insertion-related complications and mortality, length of hospital stay and costs were studied by multivariable logistic and linear regression analyses.

**Results:**

In total, 6671 patients were included, of whom 0.5% suffered major immediate insertion-related complications. Multivariable analyses, including surrogates for general morbidity, showed associations between major immediate insertion-related complications and 30-day (odds ratio 2.46 [95% CI 1.05–5.77]), 90-day (2.90 [1.35–6.21]) and 180-day (2.26 [1.05–4.83]) mortality. There were no associations between major immediate insertion-related complications and increased length of hospital stay or costs.

**Conclusion:**

This study showed that major immediate insertion-related complications, although not directly responsible for any death, were associated with increased 30-day, 90-day and 180-day mortality. These findings clearly demonstrate the importance of using all possible means to prevent avoidable insertion-related complications after central venous catheterisation.

## Introduction

Central venous catheters (CVCs) are common in modern healthcare. Unfortunately, the CVC insertion is associated with complications.^1^ Most immediate insertion-related complications are minor and do not require any treatment. However, major immediate insertion-related complications such as severe bleeding, arterial catheterisation, pneumothorax and symptomatic cardiac arrhythmia may have life-threatening consequences if not urgently treated.^2,3^ The reported incidence of immediate insertion-related complications varies between 1.1 and 34%.^1,4–11^ In our previous prospective multicentre cohort study including 12 667 CVC insertions mostly performed using ultrasound guidance, the incidence of immediate insertion-related complications was 7.7%, of which 0.4% were major complications.^12^

CVC-related infections are associated with increased patient mortality, length of hospital stay and costs,^13–16^ but data on how immediate insertion-related complications affect these outcomes are limited. A study including 480 CVC insertions in critically ill patients suggests that bleeding and pneumothorax caused by a CVC insertion have no association with mortality.^5^ Another study, including 2935 trauma patients, reported an increased intensive care unit (ICU) length of stay among patients suffering from immediate insertion-related complications, but no association with in-hospital mortality.^11^

To the best of our knowledge, no previous studies have reported an association between immediate insertion-related complications and patient mortality. Therefore, the aim of this study was to investigate whether major immediate insertion-related complications are associated with increased mortality, length of hospital stay and costs at a university hospital.

## Method

This study was approved by the Regional Ethical Review Authority, Lund, Sweden (Dnr 2018/295) on 24 April 2018. As the study was observational, the requirement of written informed consent was waived. All participants had the opportunity to opt out, which was clearly stated on advertisements at the study sites. The manuscript was written in accordance with the STROBE guidelines.^17^

### Study design

All documented non-tunnelled CVCs inserted centrally (CICCs) or femorally (FICCs) in patients ⩾16 years at Skåne University Hospital (in two locations: Lund and Malmö) between 2 March 2019 and 31 December 2020 were eligible for inclusion. Patients with CVC insertions with missing insertion date and arterial catheters accidentally recorded as CVC insertions were excluded. If a patient had multiple CVCs during the study period, only one was included based on worst case-selection, that is, the insertion with the most severe complication was chosen. If there were no complications, one insertion was chosen at random. All insertions were performed using techniques chosen by the individual operators.

### Data collection

This study was based on data collected according to the study protocol^18^ for the recently published CVC-MECH trial, which was a prospective controlled multicentre observational cohort study on immediate insertion-related complications after central venous catheterisation in the ultrasound-guided era.^12^ The participating hospitals followed the same clinical guidelines for CVC insertions, based on national recommendations.^19^ Each CVC was recorded according to a template in the electronic medical record (Melior^TM^, Cerner Corporation, North Kansas City, Missouri, USA). An automated script-based search identified and extracted all relevant variables, including patient characteristics, CVC characteristics and complication information. Data regarding patients’ previous health status (number of hospital admissions and number of outpatient visits three years before the hospital stay) and medical costs were extracted from Skåne County Council’s patient administration system. Medical records for all included patients were continuously checked for template completeness and immediate insertion-related complications by dedicated co-workers. Follow-up continued until 17 June 2021 and for deceased patients, the date of death was extracted. All variables from the different databases were merged into a main database, which was used for the statistical analyses.

### Outcomes

The outcome measures were mortality, length of hospital stay and costs. Mortality was categorised into 30-day, 90-day and 180-day mortality after insertion of the CVC. Length of hospital stay was defined as number of days in hospital (including days prior to the CVC insertion). Costs were defined as total costs for the entire hospital stay in Swedish kronor (SEK) and calculated using a template, where different departments had a specific set cost per day. Additional costs for extra measures were added based on codes in the medical journals.

### Independent variables

Minor immediate insertion-related complications (bleeding requiring external compression, self-limiting cardiac arrhythmia, arterial puncture, non-persistent nerve injury, failed catheterisation and catheter tip malposition), major immediate insertion-related complications (bleeding requiring blood transfusion, invasive intervention or with life-threatening consequences, arterial catheterisation, symptomatic cardiac arrhythmia requiring urgent medical treatment, pneumothorax and nerve injury with clinical symptoms persisting >72 h), patient sex, age, admittance to the ICU, coagulopathy, previous hospital admissions, previous outpatient visits and length of hospital stay prior to CVC insertion. Immediate insertion-related complications and their classifications are described in detail in the study protocol.^18^

### Medical record review

To assess consequences other than the predefined outcomes for patients who suffered major immediate insertion-related complications, medical records for these patients were reviewed.

### Statistical analysis

As this was a preplanned sub study to the CVC-MECH trial, for which the sample size calculation was performed, all available patients were included.^12^ To investigate associations between major immediate insertion-related complications and the outcomes, multivariable logistic regression was used for mortality and multivariable linear regression for length of hospital stay and costs. Both length of hospital stay and costs were log transformed, thus results are interpreted as percent change in hospital length of stay and percent change in costs. We used linear regression because it yields unbiased estimates of the treatment effect of major immediate insertion-related complications regardless of the underlying distribution of the outcome variable.^20^ Robust standard errors were provided for the linear regression estimates to account for potential heteroskedasticity in the error terms allowing for valid inference even when the outcome is skewed.^21,22^

Since there was a limited number of events, all independent variables could not be corrected for. The number of independent variables added to the multivariable analyses was adapted to the number of patients who had major immediate insertion-related complications and inclusion was based on univariate analyses and previous studies.^8,10,23,24^ The independent variables were added one at a time to observe how the odds ratio (OR) and beta coefficient for major immediate insertion-related complications changed.

To correct for general morbidity, the variables ‘number of previous hospital admissions’ and ‘number of previous outpatient visits’ 3 years prior to the hospital stay were chosen. As both variables exhibited a skewed distribution, they were dichotomised based on the median. Number of previous hospital admissions was dichotomised into <1 and ⩾1 visits. The number of previous outpatient visits was dichotomised into <6 and ⩾6 visits.

As the number of days from admission to CVC insertion could vary, the length of hospital stay prior to CVC insertion was compared between patients who suffered a major immediate insertion-related complication and those who did not. The Mann-Whitney *U* test was used to test if the distribution between the groups was random. In the linear regression analyses for length of hospital stay and costs, this variable was added as an independent variable.

Coagulopathy was defined as prothrombin time >1.8, activated partial thromboplastin time >1.3 × normal value (>43 s) or platelet count <50 × 10^9^/L.

In analyses including independent variables with missing data, the missing indicator method was used to avoid reduction of sample size. All missing values were replaced with ‘0’ and a missing indicator was added as an independent variable.

### Sensitivity analyses

1. To evaluate the missing data indicator method, all regression analyses where this method was used were also performed with the complete case method. If both methods reported equal results, the missing indicator method was used to avoid a reduction in sample size.
2. Since minor immediate insertion-related complications were much more common, ‘any immediate insertion-related complication’ (both minor and major) was used as an independent variable in the same regression analyses to investigate if the increased power may yield a different result.
3. In a third sensitivity analysis we used a potentially biased but more efficient estimator (Poisson regression) than our main specification (multivariable linear regression) to estimate the impact of major complications on days in hospital (not logged). No alternative method to linear regression could be identified for costs.
4. In a fourth sensitivity analysis we only included CVCs inserted with ultrasound guidance and performed the same regression analyses as in the main analyses.

SPSS Statistics version 28.0 (SPSS Inc., Chicago, IL, USA) was used for all statistical analyses. Baseline characteristics are reported with descriptive statistics, using means and standard deviations for normally distributed continuous data, and medians and interquartile ranges for skewed distributions. Numbers and percentages are used for categorical data. The results of the logistic regressions analyses are presented with OR with 95% confidence intervals (CIs) and the results of the linear regressions are presented as beta coefficients with 95% CI. A *p*-value <0.05 was considered statistically significant.

## Results

**Figure 1.**
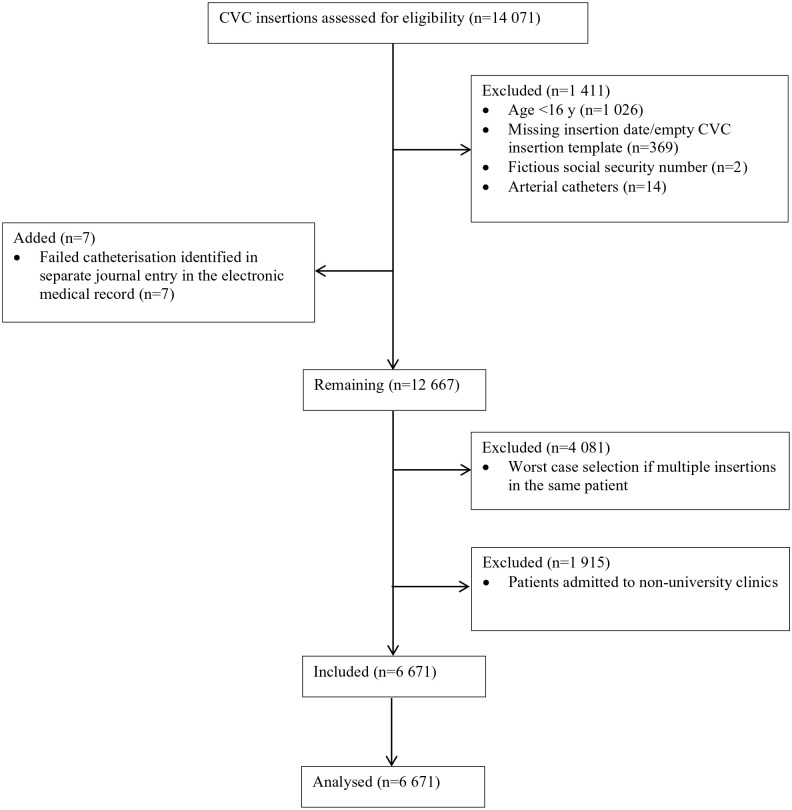
Consort diagram. CVC: central venous catheter.

The flow chart in Figure 1 describes details of the patient inclusion. After removing patients admitted to non-university clinics and applying worst-case selection on patients who received multiple CVCs, 6671 unique patients were included. The CVC insertions were performed by 210 individual operators. Baseline patient characteristics are presented in Table 1. CVC insertion characteristics, operator experience and number of CVC insertions per hospital are presented in Table 2.

**Table 1.** Patient baseline characteristics.

Patients, *n*	6671
Female sex, *n* (%)	2660 (40)
Male sex, *n* (%)	4011 (60)
Age (y), mean ± SD	65 ± 15
BMI (kg/m^2^), mean ± SD	27 ± 5.6
Admittance to the intensive care unit, *n* (%)	2513 (38)
Coagulopathy,^1^ *n* (%)	897 (13)
Invasive positive pressure ventilation, *n* (%)	4241 (64)
Previous hospital admissions,^2^ *n* (%)	
<1	3208 (48)
⩾1	2273 (34)
Missing	1190 (18)
Previous outpatient visits,^3^ *n* (%)	
<6	2668 (40)
⩾6	2813 (42)
Missing	1190 (18)
Indication,^4^ *n* (%)	
Infusion of irritant substances	2423 (36)
Blood sampling	2006 (30)
Heart surgery	1898 (28)
Haemodynamic monitoring	1747 (26)
Inadequate peripheral access	1300 (19)
Fluid resuscitation	1180 (18)
Parenteral nutrition	1100 (16)
Introducer	221 (3.3)
Other	203 (3.0)
Dialysis	173 (2.6)
Plasmapheresis	9 (0.1)

1 Prothrombin time >1.8, activated partial thromboplastin time >1.3 × normal value (>43 s) or platelet count <50 × 10^9^/L.

2 Number of hospital admissions three years prior to the hospital stay.

3 Number of outpatient visits three years prior to the hospital stay.

4 Some patients had multiple indications.

**Table 2.** CVC insertion characteristics, operator experience and CVC insertions per hospital.

CVC insertions, *n*	6671
Insertion at night,^1^ *n* (%)	814 (12)
Vein for insertion, *n* (%)	
Right internal jugular vein	4976 (75)
Right subclavian/axillary vein^2^	847 (13)
Left internal jugular vein	423 (6.3)
Left subclavian/axillary vein^2^	199 (3)
Right femoral vein^3^	45 (0.7)
Right external jugular vein	35 (0.5)
Left femoral vein^3^	19 (0.3)
Left external jugular vein	7 (0.1)
Missing	120 (1.8)
Insertion technique, *n* (%)	
Ultrasound guidance	6297 (94)
Out-of-plane	4354 (65)
In-plane	1563 (23)
US before insertion only	211 (3.2)
In-plane/out-of-plane	169 (2.5)
No ultrasound guidance	215 (3.2)
Landmark	200 (3.0)
Change over wire	15 (0.2)
Missing	159 (2.4)
CVC bore size, *n* (%)	
<9 Fr	4626 (69)
⩾9 Fr	1069 (16)
Missing	976 (15)
Operator experience,^4^ *n* (%)	
<100 catheterisations	1351 (20)
⩾100 catheterisations	5005 (75)
Missing	315 (4.7)
Hospital site, *n* (%)	
Lund	4365 (65)
Malmö	2306 (35)

1 Between 21:00 and 07:00.

2 Infraclavicular approach. No data regarding the exact puncture site.

3 Common or superficial femoral vein, there is no data regarding the exact puncture site.

4 In total 210 operators performed all CVC-insertions. Estimated number of individual CVC insertions per insertion site, prior to study start.

A total of 516 (7.7%) patients experienced immediate insertion-related complications, of which 36 (0.5%) were major and 490 (7.3%) were minor. The most common minor complications were bleeding requiring external compression (215 cases; 3.2%) and catheter tip malposition (186 cases; 2.8%). The most common major complications were pneumothorax (14 cases; 0.2%) and arterial catheterisation (9 cases; 0.1%).

There was no difference in length of hospital stay prior to the CVC insertion between patients with or without a major immediate insertion-related complication (*p* = 0.419, 55 patients had missing data). This indicated a distribution at random for this variable and justified analyses regarding length of hospital stay from the start of the admittance. For 1190 patients, the data extracted from Skåne County Council’s patient administration system were randomly unavailable. Therefore, those patients had missing data for ‘number of previous hospital admissions’ and ‘number of previous outpatient visits’. The missing indicator method was used for these variables. Univariate analyses of independent variables and the outcome measures are reported in Supplemental Tables S1 and S2.

### Mortality analyses

Of the 36 patients who suffered major immediate insertion-related complications, 7 (19%) died within 30 days and 10 (28%) within 90 days. At 180 days, the mortality remained unchanged. Of the 6 635 patients who did not have a major immediate insertion-related complication, 657 (9.9%) died within 30 days, 875 (13%) within 90 days and 1061 (16%) within 180 days.

**Figure 2.**
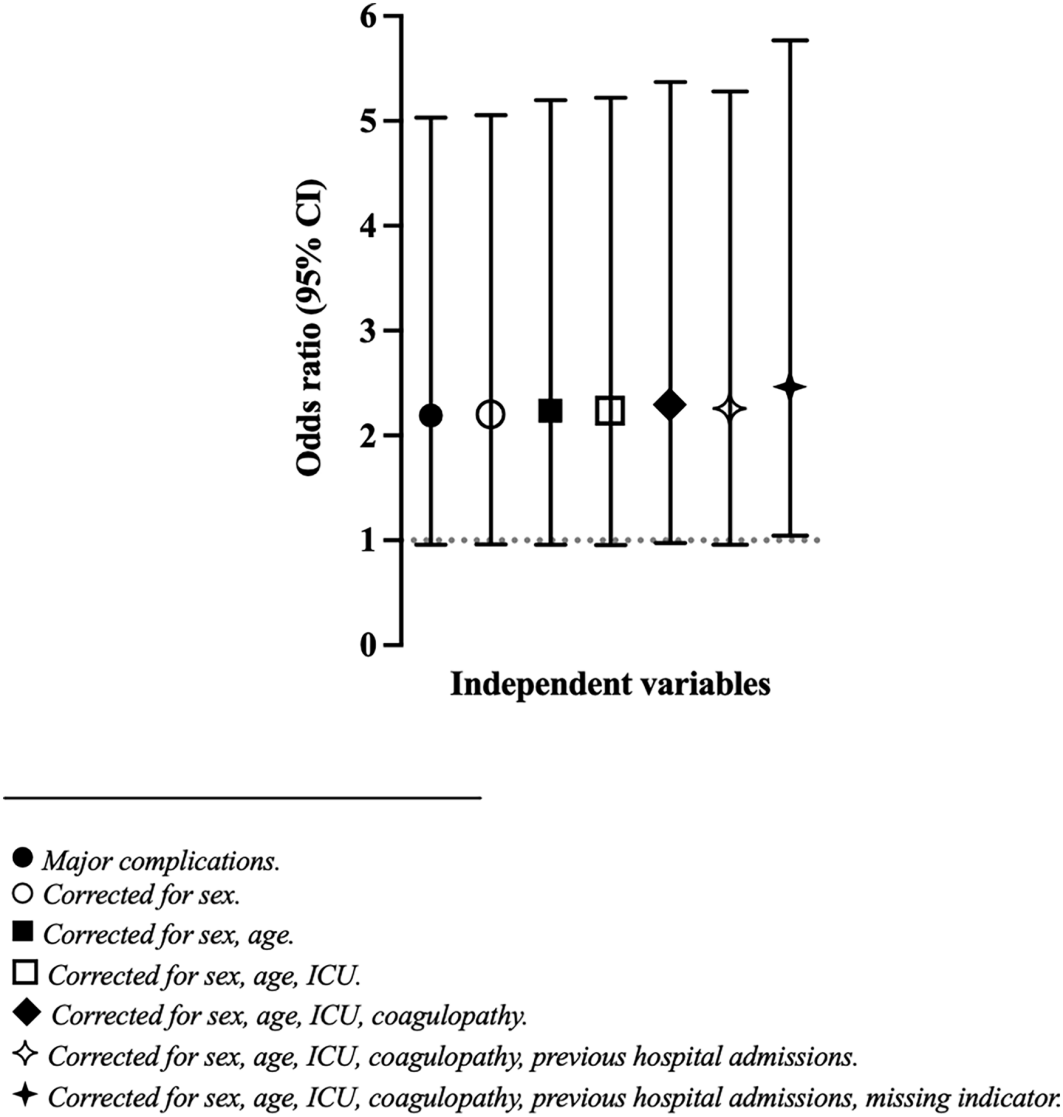
Cumulative estimates of odds ratio on 30-day mortality when adding independent variables stepwise in logistic regression analysis.

The results from the multivariable logistic regression analyses for mortality are reported in Table 3. Major immediate insertion-related complications were associated both with 30-day (OR 2.46 [95% CI 1.05 to 5.77], *p* = 0.039), 90-day (2.90 [1.35 to 6.21], *p* = 0.006) and 180-day (2.26 [1.05 to 4.83], *p* = 0.037) mortality. Figure 2 shows how the OR with 95% CI for major immediate insertion-related complications changed with stepwise addition of the independent variables to the 30-day mortality analysis.

**Table 3.** Logistic regression analysis for mortality.

	Odds ratio with 95% confidence intervals					
	30-Day mortality^1^	*p*-Value	90-Day mortality^1^	*p*-Value	180-Day mortality^1^	*p*-Value
Major complication	2.46 (1.05 to 5.77)	0.039	2.90 (1.35 to 6.21)	0.006	2.26 (1.05 to 4.83)	0.037

1 Corrected for sex, age, admittance to intensive care unit y/n, coagulopathy (defined as prothrombin time >1.8, activated partial thromboplastin time >1.3 × normal value (>43 s) or platelet count <50 × 10^9^/L), number of previous hospital admissions (4 years prior to the hospital stay) dichotomised and a missing indicator. All 6671 cases in analysis.

### Length of hospital stay and cost analyses

The median length of hospital stay was 18 days [IQR 8.6 to 36] for patients who suffered major immediate insertion-related complications compared to 13 days [IQR 8.1 to 23] for those who did not. For patients who suffered major immediate insertion-related complications, the median cost was 180,000 Swedish crowns (SEK) [IQR 81,500 to 439,500], and for those who did not, 193,000 SEK [IQR 107,000 to 288,800].

The results from the multivariable linear regression analyses for length of hospital stay and costs are reported in Table 4. The linear regression analyses showed a beta coefficient of 0.20 (95% CI −0.11 to 0.50, *p* = 0.207) for log-transformed length of hospital stay and a beta coefficient of 0.04 (95% CI −0.42 to 0.50, *p* = 0.871) for log transformed costs. This is equivalent to a 22% increase in length of hospital stay and 3.9% increase in costs, although not statistically significant.

**Table 4.** Linear regression analyses with robust standard errors for length of hospital stay and costs.

	Beta coefficient with 95% confidence intervals			
	Length of hospital stay^1^	*p*-Value	Costs^2^	*p*-Value
Major complication	0.20 (−0.11 to 0.50)	0.207	0.04 (−0.42 to 0.50)	0.871

1 Log transformed. Corrected for sex, age, admittance to intensive care unit y/n, coagulopathy (defined as prothrombin time >1.8, activated partial thromboplastin time >1.3 × normal value (>43 s) or platelet count <50 × 10^9^/L), number of previous hospital admissions (3 years prior to the hospital stay) dichotomised, number of previous outpatient visits (3 years prior to the hospital stay) dichotomised, a missing indicator and length of hospital stay prior to CVC insertion. In total, 6593/6 671 (99%) cases in analysis.

2 Log transformed. Corrected for sex, age, admittance to intensive care unit y/n, coagulopathy (defined as prothrombin time >1.8, activated partial thromboplastin time >1.3 × normal value (>43 s) or platelet count <50 × 10^9^/L), number of previous hospital admissions (3 years prior to the hospital stay) dichotomised, number of previous outpatient visits (3 years prior to the hospital stay) dichotomised and length of hospital stay prior to CVC insertion. In total, 5291/6671(79%) cases in analysis.

### Sensitivity analyses

1. The results of the complete case analysis method and the missing indicator method were similar (Supplemental Tables S3 and S4).
2. Any immediate insertion-related complications were associated with 30-day, 90-day and 180-day mortality (Supplemental Tables S5 and S6).
3. Poisson regression yielded a slightly lower estimate than the linear regression model on length of hospital stay, suggesting that the Poisson estimates have a downwards bias towards zero (Supplemental Table S7). However, Poisson regression yielded much smaller standard errors, which suggests a significant positive impact of severe immediate insertion-related complications on the length of hospital stay.
4. The regression analyses where only CVCs inserted with ultrasound guidance were included showed similar results as the main analyses (Supplemental Tables S8 and S9).

### Consequences of major immediate insertion-related complications

Although many patients suffered severe consequences (such as delayed treatment, deterioration, and requirement of invasive interventions) due to major immediate insertion-related complications, no patients were judged to have died due to the major complication. For details, please see Supplemental Data S1 and Table S10.

## Discussion

This prospective observational study including 6671 patients showed that major immediate insertion-related complications after central venous catheterisation were associated with increased 30-day, 90-day and 180-day mortality.

The incidence of major immediate insertion-related complications in the present study (0.5%) is nearly the same as in our previous retrospective analysis from multiple hospitals in the same region (which included the hospital in this study).^10^ This can be compared to the 3SITES trial,^25^ in which the major complication rate was 1.4%. Ultrasound guidance, which is known to reduce the number of immediate insertion-related complications,^26–29^ was used in 43% of the CVC insertions in the 3SITES trial compared to 94% in the present cohort, which may explain our lower incidence of major complications.

Some methodological issues deserve mention. We demonstrated an association between major immediate insertion-related complications and 30-day mortality. To test the robustness of the model, confounders were added one at a time to give a sense to how important potential unmeasured confounding may be. As the odds ratio with 95% CI did not change substantially, this suggests that the model is fairly robust. It should also be noted that it is uncommon for large public health data sets to include data on severity of illness. To address this important aspect of a mortality analysis, we included the number of ‘previous hospital admissions’ and the number of ‘previous outpatient visits’ as independent variables in the regression models.

We used linear regression with robust standard errors in the main analyses and demonstrated a 22% increase in length of hospital stay for patients with major immediate insertion-related complications, although none of them were statistically significant. In the sensitivity analyses ‘any immediate insertion-related complication’ was used instead of ‘major immediate insertion-related complications’ and although the point estimate for length of hospital stay was decreased to 9.2%, the association was significant, probably due to the increased power. Furthermore, whilst linear regression yields unbiased estimates of the true impact of major immediate insertion-related complications, it may be more efficient to use a Poisson regression if the true underlying functional form indeed follows a Poisson distribution. We tested estimating the impact of major complications on hospital days using Poisson regression and found that the estimates differed from the linear regression suggesting a small downwards bias in Poisson regression. Moreover, the standard errors were much smaller using Poisson regression to the extent that even the slightly downwards biased estimate of Poisson regression was statistically significant. This suggests that major immediate insertion-related complications not only have meaningfully large healthcare system impacts but also that this impact is statistically significant.

Available studies have been unable to find associations between immediate insertion-related complications after CVC insertion and mortality.^5,11^ Compared to those studies, the present study had greater power, investigated longer term mortality (up to 180 days after CVC insertion) and also corrected for patient morbidity. None of the major immediate insertion-related complications in the present study were deemed directly responsible for any deaths. Nevertheless, a strong association with mortality was demonstrated. The reason for this is not explained by this study, but one hypothesis is that major immediate insertion-related complications can delay treatment or require invasive interventions, which in turn could affect mortality.

As demonstrated in Figure 2, the OR for 30-day mortality was unchanged when independent variables were added stepwise. The second set of sensitivity analyses, in which ‘major immediate insertion-related complications’ were changed to ‘any immediate insertion-related complication’, also confirmed the association with mortality. These findings indicate that the results in the present study are robust.

This study has several limitations. First, the study relies on a strong tradition to document every CVC insertion in the medical records, but still some insertions may remain undocumented. Second, we aimed to investigate clinical practice in an ultrasound-guided era. Ultrasound guidance was not used in 3% of the cases, but when those were excluded in sensitivity analyses, the results remained very similar. Third, we lack data regarding the exact puncture site for CVCs inserted in the subclavian/axillary veins. Fourth, due to unavailable economic data for patients admitted to non-university clinics, only patients admitted to a university hospital were included. Fifth, although the independent variables were selected very carefully, unmeasured confounders may remain. Sixth, although the missing indicator method was used and found valid, missing values may still have affected the results. Seventh, only the costs immediately relevant to the hospital stay were included in this study. Other costs to the individual, their family, society and wider health-care systems such as later prescriptions and follow up visits, were not considered. Eighth, there were few patients with major immediate insertion-related complications, yielding a less precise result with wide confidence intervals and a risk of a limited goodness of fit. However, when power was increased in the sensitivity analyses, the association with mortality remained.

In conclusion, this study showed that major immediate insertion-related complications after central venous catheterisation were associated with increased 30-day, 90-day and 180-day mortality. To the best of our knowledge these findings are new and demonstrate the importance of avoiding insertion-related complications as they are associated with serious consequences for the patients.

## Supplemental Material

sj-pdf-1-jva-10.1177_11297298231222929 – Supplemental material for Major immediate insertion-related complications after central venous catheterisation and associations with mortality, length of hospital stay, and costs: A prospective observational study Supplemental material, sj-pdf-1-jva-10.1177_11297298231222929 for Major immediate insertion-related complications after central venous catheterisation and associations with mortality, length of hospital stay, and costs: A prospective observational study by Sofia Ingefors, Maria Adrian, Gawain Heckley, Ola Borgquist and Thomas Kander in The Journal of Vascular Access
