# Supplementary material for: Major immediate insertion-related complications after central venous catheterisation and associations with mortality, length of hospital stay, and costs: A prospective observational study
Source: J Vasc Access. 2024 Jan 24;26(2):487–96. doi: 10.1177/11297298231222929 (PMC11894852; doi:10.1177/11297298231222929)
Supplement: sj-pdf-1-jva-10.1177_11297298231222929 – Supplemental material for Major immediate insertion-related complications after central venous catheterisation and associations with mortality, length of hospital stay, and costs: A prospective observational study [file sj-pdf-1-jva-10.1177_11297298231222929.pdf]

Major immediate insertion-related complications after central venous catheterisation and associations with mortality, length of hospital stay, and costs: a prospective observational study

Supplementary Material

Table of contents

Supplementary Table S1. Univariate logistic regression analyses for mortality. 2

Supplementary Table S2. Univariate linear regression analyses for length of hospital stay and costs. 3

Supplementary Table S3. Logistic regression analysis for mortality (complete case analysis method). 4

Supplementary Table S4. Linear regression analyses with robust standard errors for length of hospital stay and costs (complete case analysis method). 5

Supplementary Table S5. Logistic regression analysis for mortality ("Any immediate insertion-related complication"). 6

Supplementary Table S6. Linear regression analyses with robust standard errors for length of hospital stay and costs ("Any immediate insertion-related complication"). 7

Supplementary Table S7. Linear regression analysis with robust standard errors and Poisson regression analysis for length of hospital stay (not logged). 8

Supplementary Table S8. Logistic regression analysis for mortality (only including CVCs inserted with ultrasound guidance). 9

Supplementary Table S9. Linear regression analyses with robust standard errors for length of hospital stay and costs (only including CVCs inserted with ultrasound guidance) 10

Supplementary Data S1 11

Supplementary Table S10. Very severe consequences of the major immediate insertion-related complications per type of complication. 13

Supplementary Table S1. Univariate logistic regression analyses for mortality.

**Total number of patients, n=6 671**	**Odds Ratio with 95% Confidence Intervals.**					
	30-day mortality n=6 671	P-value	90-day mortality n=6 671	P-value	180-day mortality n=6 671	P-value
**Female sex**	1.11 (0.95 to 1.31)	0.203	1.18 (1.03 to 1.36)	**0.022**	1.28 (1.12 to 1.46)	**<0.001**
**Age**	1.03 (1.03 to 1.04)	**<0.001**	1.04 (1.03 to 1.05)	**<0.001**	1.04 (1.03 to 1.04)	**<0.001**
**Admittance to the ICU**	1.59 (1.35 to 1.86)	**<0.001**	1.16 (1.01 to 1.34)	**0.040**	0.91 (0.79 to 1.04)	0.159
**Coagulopathy**[a]	1.83 (1.50 to 2.24)	**<0.001**	1.76 (1.47 to 2.11)	**<0.001**	1.65 (1.39 to 1.96)	**<0.001**
**Previous hospital admissions**[b]	1.07 (0.90 to 1.26)	0.450	1.16 (1.00 to 1.34)	0.053	1.29 (1.12 to 1.47)	**<0.001**
**Previous out-patient visits**[c]	0.93 (0.79 to 1.09)	0.363	0.98 (0.85 to 1.14)	0.82	1.08 (0.95 to 1.24)	0.240

[a] *Prothrombin time >1.8, activated partial thromboplastin time >1.3 × normal value (>43 s) or platelet count <50 × 10^9/L.*
[b] *Number of hospital admissions three years prior to the hospital stay, dichotomised.*
[c] *Number of out-patient visits three years prior to the hospital stay, dichotomised.*

Supplementary Table S2. Univariate linear regression analyses for length of hospital stay and costs.

**Total number of patients, n=6 671**	**Beta Coefficient with 95% Confidence Intervals.**			
	Length of hospital stay[a] n=6 611	P-value	Costs[b] n=5 302	P-value
**Female sex**	-0.023 (-0.064 to 0.018)	0.275	-0.088 (-0.145 to -0.032)	**0.002**
**Age**	0.000 (-0.001 to 0.002)	0.522	-0.005 (-0.006 to -0.003)	**<0.001**
**Admittance to the ICU**	-0.140 (-0.182 to -0.098)	**<0.001**	0.039 (-0.018 to 0.096)	0.181
**Coagulopathy[c]**	0.099 (0.040 to 0.158)	**0.001**	0.023 (-0.058 to 0.103)	0.580
**Previous hospital admissions[d]**	0.027 (-0.016 to 0.069)	0.222	-0.111 (-0.167 to -0.055)	**<0.001**
**Previous out-patient visits[e]**	0.036 (-0.005 to 0.076)	0.089	-0.030 (-0.085 to 0.025)	0.284
**Length of hospital stay prior to CVC insertion**	0.003 (0.002 to 0.003)	**<0.001**	-0.002 (-0.003 to -0.001)	<0.001

[a] *Log transformed*
[b] *Log transformed.*
[c] *Prothrombin time >1.8, activated partial thromboplastin time >1.3 × normal value (>43 s) or platelet count <50 × 10^9/L.*
[d] *Number of hospital admissions three years prior to the hospital stay, dichotomised.*
[e] *Number of out-patient visits three years prior to the hospital stay, dichotomised.*

Supplementary Table S3. Logistic regression analysis for mortality (complete case analysis method).

	Odds Ratio with 95% Confidence Intervals.					
	30-day mortality[a]	p-value	90-day mortality[a]	p-value	180-day mortality[a]	p-value
**Major complication**	2.46 (1.05 to 5.77)	**0.039**	2.88 (1.35 to 6.16)	**0.006**	2.24 (1.05 to 4.78)	**0.038**

[a] *Corrected for sex, age, admittance to intensive care unit y/n, coagulopathy (defined as prothrombin time >1.8, activated partial thromboplastin time >1.3 × normal value (>43 s) or platelet count <50 × 10^9/L), number of previous hospital admissions (three years prior to the hospital stay) dichotomised (complete case analysis method; missing is kept as missing). In total, 5 481/ 6 671 (82%) cases in analysis.*

Supplementary Table S4. Linear regression analyses with robust standard errors for length of hospital stay and costs (complete case analysis method).

	**Beta Coefficient with 95% Confidence Intervals.**			
	Length of hospital stay[a]	p-value	Costs[b]	p-value
**Major complication**	0.18 (-0.11 to 0.50)	0.209	0.04 (-0.42 to 0.50)	0.871

---

[a] *Log transformed. Corrected for sex, age, admittance to intensive care unit y/n, coagulopathy (defined as prothrombin time >1.8, activated partial thromboplastin time >1.3 × normal value (>43 s) or platelet count <50 × 10^9/L), number of previous hospital admissions (three years prior to the hospital stay) dichotomised (complete case analysis method; missing is kept as missing), number of previous out-patient visits (three years prior to the hospital stay) dichotomised (complete case analysis method; missing is kept as missing) and length of hospital stay prior to CVC insertion. In total, 5 459/6 671 (82%) cases in analysis.*

[b] *Log transformed. Corrected for sex, age, admittance to intensive care unit y/n, coagulopathy (defined as prothrombin time >1.8, activated partial thromboplastin time >1.3 × normal value (>43 s) or platelet count <50 × 10^9/L), number of previous hospital admissions (three years prior to the hospital stay) dichotomized (complete case analysis method; missing is kept as missing), number of previous out-patient visits (three years prior to the hospital stay) dichotomized (complete case analysis method; missing is kept as missing) and length of hospital stay prior to CVC insertion. In total, 5 291/6 671(79%) cases in analysis.*

Supplementary Table S5. Logistic regression analysis for mortality ("Any immediate insertion-related complication").

	**Odds Ratio with 95% Confidence Intervals.**					
	30-day mortality[a]	p-value	90-day mortality[a]	p-value	180-day mortality[a]	p-value
**Any complication**	1.67 (1.28 to 2.17)	**<0.001**	1.82 (1.44 to 2.29)	**<0.001**	1.70 (1.36 to 2.13)	**<0.001**

[a] *Corrected for sex, age, admittance to intensive care unit y/n, coagulopathy (defined as prothrombin time >1.8, activated partial thromboplastin time >1.3 × normal value (>43 s) or platelet count <50 × 10^9/L), number of previous hospital admissions (three years prior to the hospital stay) dichotomised and a missing indicator. All 6 671 cases in analysis.*

Supplementary Table S6. Linear regression analyses with robust standard errors for length of hospital stay and costs ("Any immediate insertion-related complication").

	**Beta Coefficient with 95% Confidence Intervals.**			
	Length of hospital stay[a]	p-value	Costs[b]	p-value
**Any complication**	0.09 (0.01 to 0.17)	**0.031**	-0.07 (-0.18 to 0.05)	0.234

[a] *Log transformed. Corrected for sex, age, admittance to intensive care unit y/n, coagulopathy (defined as prothrombin time >1.8, activated partial thromboplastin time >1.3 × normal value (>43 s) or platelet count <50 × 10^9/L), number of previous hospital admissions (three years prior to the hospital stay) dichotomised, number of previous out-patient visits (three years prior to the hospital stay) dichotomised, a missing indicator and length of hospital stay prior to CVC insertion. In total, 6 593/6 671 (99%) cases in analysis.*

[b] *Log transformed. Corrected for sex, age, admittance to intensive care unit y/n, coagulopathy (defined as prothrombin time >1.8, activated partial thromboplastin time >1.3 × normal value (>43 s) or platelet count <50 × 10^9/L), number of previous hospital admissions (three years prior to the hospital stay) dichotomised, number of previous out-patient visits (three years prior to the hospital stay) dichotomised and length of hospital stay prior to CVC insertion. In total, 5 291/6 671(79%) cases in analysis.*

Supplementary Table S7. Linear regression analysis with robust standard errors and Poisson regression analysis for length of hospital stay (not logged).

	**Beta Coefficient with 95% Confidence Intervals.**			
	Linear regression for length of hospital stay[a]	p-value	Poisson regression for length of hospital stay[a]	p-value
**Major complication**	2.30 (-2.93 to 7.52)	0.388	1.20 (1.12 to 1.29)	<0.001

[a] *Corrected for sex, age, admittance to intensive care unit y/n, coagulopathy (defined as prothrombin time >1.8, activated partial thromboplastin time >1.3 × normal value (>43 s) or platelet count <50 × 10^9/L), number of previous hospital admissions (three years prior to the hospital stay) dichotomised, number of previous out-patient visits (three years prior to the hospital stay) dichotomised, a missing indicator and length of hospital stay prior to CVC insertion. Poisson regression coefficient is exponentiated and interpreted in terms of days in hospital and comparable to OLS estimate. In total, 6 593/6 671 (99%) cases in analysis.*

Supplementary Table S8. Logistic regression analysis for mortality (only including CVCs inserted with ultrasound guidance).

	**Odds Ratio with 95% Confidence Intervals**					
	30-day mortality[a]	p-value	90-day mortality[a]	p-value	180-day mortality[a]	p-value
**Major complication**	2.53 (1.07 to 5.97)	**0.034**	2.98 (1.38 to 6.42)	**0.005**	2.30 (1.07 to 4.95)	**0.034**

[a] *Corrected for sex, age, admittance to intensive care unit y/n, coagulopathy (defined as prothrombin time >1.8, activated partial thromboplastin time >1.3 × normal value (>43 s) or platelet count <50 × 10^9/L), number of previous hospital admissions (three years prior to the hospital stay) dichotomised and a missing indicator. In total 6 297/6 671 (94%) cases in analysis.*

Supplementary Table S9. Linear regression analyses with robust standard errors for length of hospital stay and costs (only including CVCs inserted with ultrasound guidance)

	**Beta Coefficient with 95% Confidence Intervals**			
	Length of hospital stay[a]	p-value	Costs[b]	p-value
**Major complication**	0.21 (-0.11 to 0.52)	0.198	0.04 (-0.44 to 0.52)	0.869

[a] *Log transformed. Corrected for sex, age, admittance to intensive care unit y/n, coagulopathy (defined as prothrombin time >1.8, activated partial thromboplastin time >1.3 × normal value (>43 s) or platelet count <50 × 10^9/L), number of previous hospital admissions (three years prior to the hospital stay) dichotomised, number of previous outpatient visits (three years prior to the hospital stay) dichotomised, a missing indicator and length of hospital stay prior to CVC insertion. In total, 6 232/6 671 (93%) cases in analysis.*

[b] *Log transformed. Corrected for sex, age, admittance to intensive care unit y/n, coagulopathy (defined as prothrombin time >1.8, activated partial thromboplastin time >1.3 × normal value (>43 s) or platelet count <50 × 10^9/L), number of previous hospital admissions (three years prior to the hospital stay) dichotomised, number of previous outpatient visits (three years prior to the hospital stay) dichotomised and length of hospital stay prior to CVC insertion. In total, 5 007/6 671(75%) cases in analysis.*

Supplementary Data S1

Consequences of major immediate insertion-related complications

To assess consequences of major immediate insertion-related complications, medical records were reviewed. All major immediate insertion-related complications were further divided into having caused a very severe consequence or not. A very severe consequence was defined as a major immediate insertion-related complication that required invasive intervention, causing deterioration or delayed treatment. If a pneumothorax required a chest tube or caused deterioration of the patient, if a bleeding or an arterial catheterisation required vascular intervention or transfusion, if an arrhythmia was symptomatic or life-threatening or required urgent medical intervention, or if a nerve injury did not heal after several months, the complication was considered very severe.

Results of the medical record review

None of the patients with major complications were judged to have died as a direct consequence of the major complication. Very severe consequences of the major immediate insertion-related complications are reported in Table d. Major immediate insertion-related complications occurred in 36 patients, and 18 (50%) of those suffered very severe consequences. In total 14 patients were diagnosed with pneumothorax, whereof eight spontaneously recovered. Six patients required a chest tube, of which two suffered from a complication associated with the chest tube, including a haemothorax causing deterioration in one patient and subcutaneous emphysema leading to a two-day discharge delay in another patient. One of these patients required both chest tube insertion and intubation due to life-threatening worsening of an already failing lung function.

In total 15 patients were diagnosed with bleeding grade 3-4 or arterial catheterisation. Of these, eight recovered by simple interventions like compression, local anaesthesia with adrenaline, or tranexamic acid. One patient required monitoring in the post-operative ward due to carotid artery catheterisation and subsequent risk to develop an airway-threatening haematoma. Six patients experienced very severe consequences. Five required transfusions due to decreasing haemoglobin, of which one

required suturing by a vascular surgeon to stop the bleeding. One of the patients requiring transfusion suffered a life-threatening hematoma on the neck, which possibly delayed a computed tomography (CT) of the head CT and a CT pulmonary angiography after bilateral pulmonary embolism and cardiac arrest. One patient required vascular intervention and had elective surgery delayed 42 days.

Six patients suffered from arrhythmias grade 3-4 and these were all characterised as very severe consequences. One patient suffered a ventricular tachycardia treated with defibrillation and amiodarone. One patient suffered a sinus arrest during a TAVI procedure which required transvenous pacing. Four patients suffered supraventricular tachycardias, of which three were treated with magnesium and fluids, one of these additionally with metoprolol. The fourth patient had a symptomatic arrhythmia with a decrease in blood pressure but recovered spontaneously.

One patient suffered from a persistent nerve injury, defined as clinical signs of neurological dysfunction persisting more than 72 hours. The patient regained full sensibility 4 months later.

Supplementary Table S10. Very severe consequences of the major immediate insertion-related complications per type of complication.

Type of major immediate insertion-related complication	Patients with very severe consequences of the major immediate insertion-related complications, n (% of total per type of complication)	Total
**Pneumothorax**	6 (43)	14
**Bleeding grade 3-4[a] /arterial catheterisation**	6 (40)	15
**Arrhythmia grade 3-4[b]**	6 (100)	6
**Persistent nerve injury[c]**	0 (0)	1

[a]*Bleeding requiring blood transfusion, invasive intervention or with life-threatening consequences.*
[b]*Symptomatic or life-threatening arrhythmia requiring urgent medical intervention.*
[c]*Nerve injury with clinical signs persisting >72h.*
